# Supplementary material for: High-Content Drug Discovery Targeting Molecular Bladder Cancer Subtypes
Source: Int J Mol Sci. 2022 Sep 14;23(18):10605. doi: 10.3390/ijms231810605 (PMC9506379; doi:10.3390/ijms231810605)
Supplement: Supplementary file 1 [file ijms-23-10605-s001.zip › ijms-1905819-supplementary.pdf]

Supplementary Information

High-content drug discovery targeting molecular bladder cancer subtypes

*Sébastien Rinaldetti[1,2], Qiong Zhou[1], Joshua M. Abbott[1], Florus C. DeJong[3], Hector Esquer[1], James C. Costello[4,6], Dan Theodorescu[5], and Daniel V. LaBarbera[1,6,7,]**

1. The Skaggs School of Pharmacy and Pharmaceutical Sciences, Department of Pharmaceutical Sciences, The University of Colorado Anschutz Medical Campus, Aurora, Colorado, USA
2. The Department of Hematology-oncology, Centre Hospitalier de Luxembourg, Luxembourg City, Luxembourg
3. The Department of Urology, Erasmus MC Cancer Institute, Rotterdam, The Netherlands
4. Department of Pharmacology, University of Colorado Anschutz Medical Campus, Aurora, Colorado, USA
5. Cedars-Sinai Samuel Oschin Comprehensive Cancer Institute, Los Angeles, California, USA
6. The University of Colorado Cancer Center, University of Colorado Anschutz Medical Campus, Aurora, Colorado, USA
7. The Center for Drug Discovery, University of Colorado Anschutz Medical Campus, Aurora, Colorado, USA

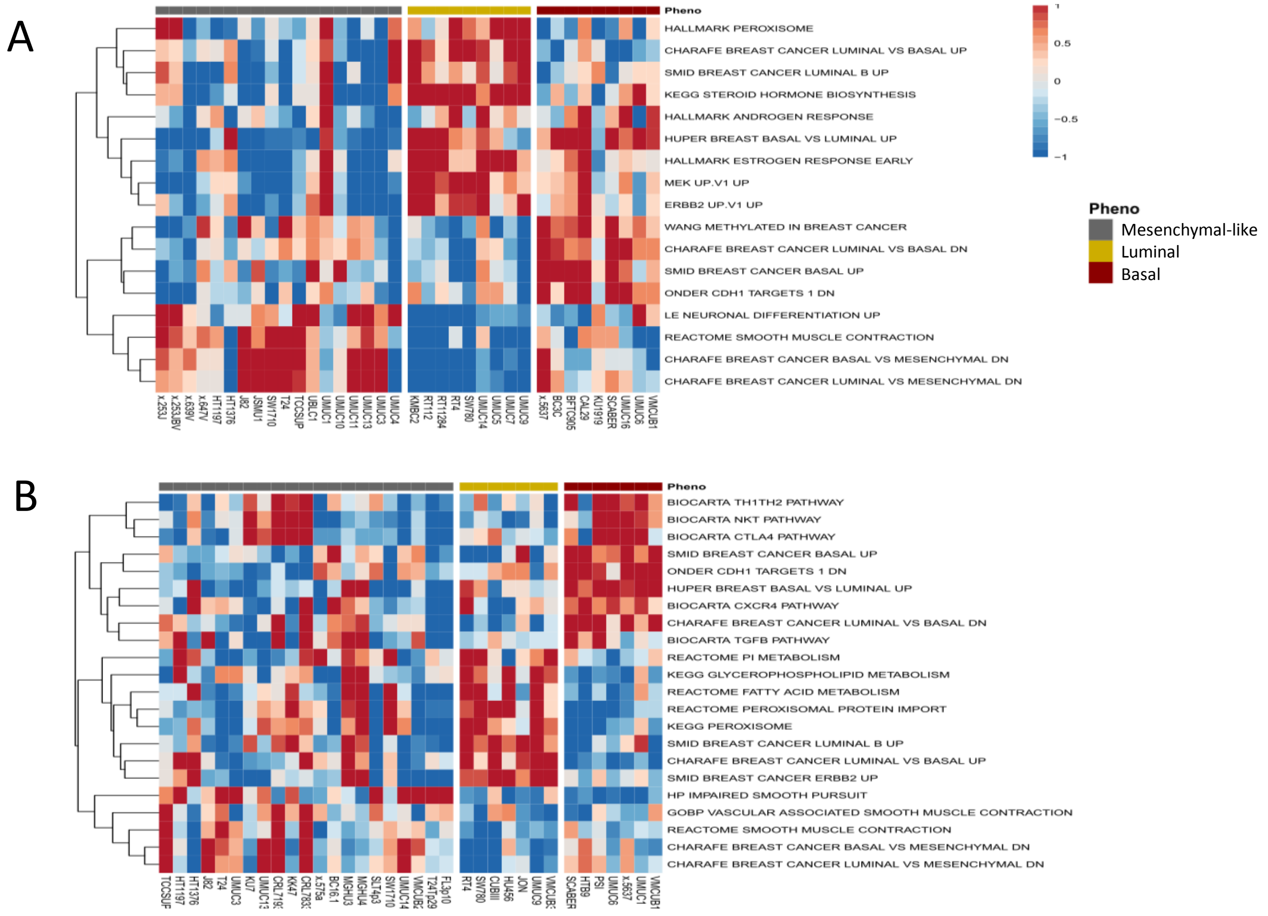

Figure S1. (A) Heatmap plotting relevant enriched gene signatures within the CCLE cell lines. **(B)** Heatmap plotting relevant enriched gene signatures within the BLA-40 cell lines.

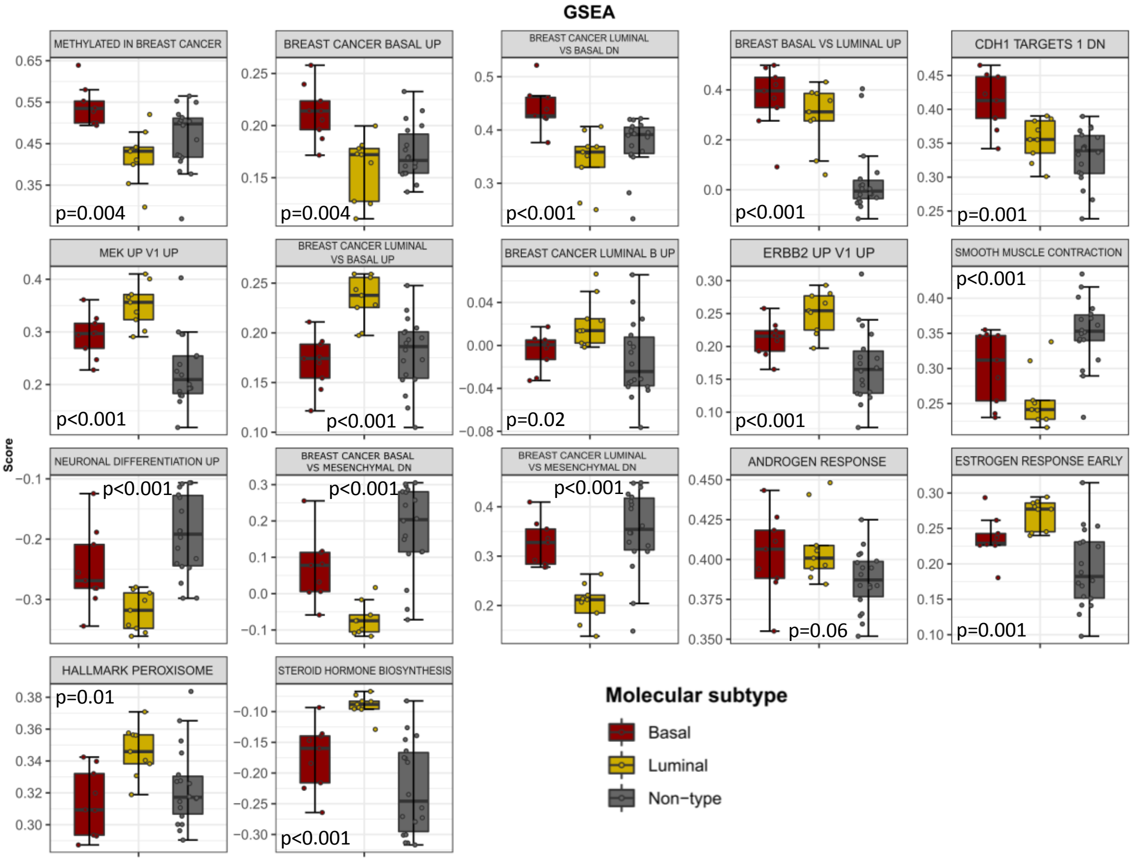

Figure S2. Boxplots of relevant enriched gene signatures within the CCLE cell lines (n=36).

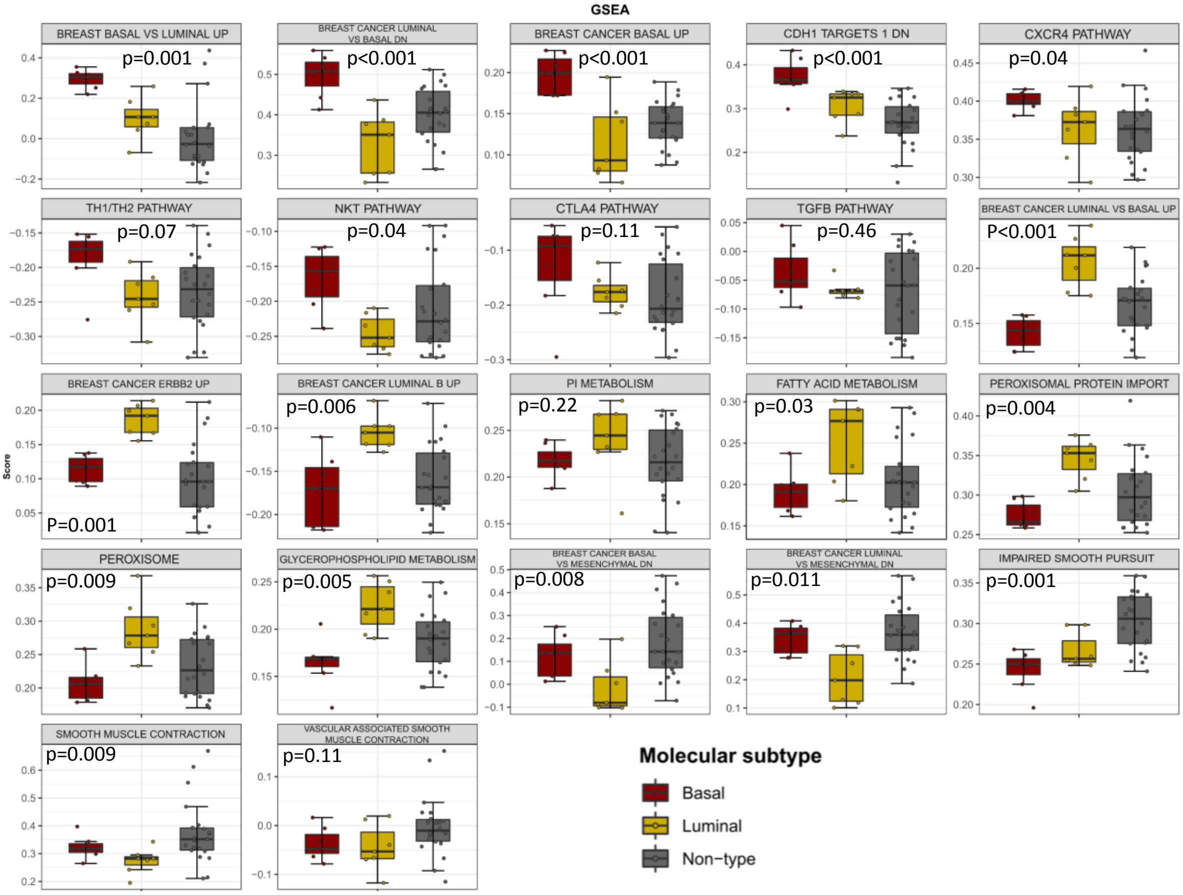

Figure S3. Boxplots of relevant enriched gene signatures within the BLA-40 cell lines (n=36).

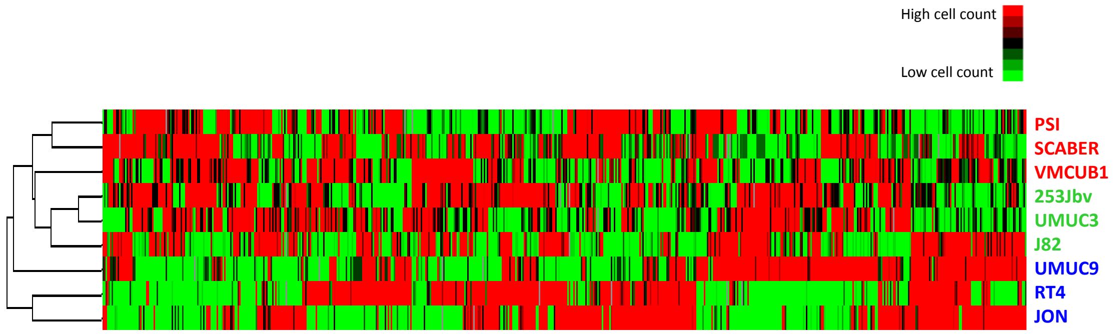

Figure S4. Unsupervised clustering of 9 cell lines based on their response to 616 inhibitors. Drug response was assessed by cell count after 72h incubation with 10μM of each drug. Blue = luminal, Red = basal, green = mesenchymal-like

BASAL	p-value	LUMINAL	p-value	MESENCHYMAL-LIKE	p-value
**Citarinostat (ACY-241)**	0.002	**MK-886 (L-663.536)**	0.007	**SB273005**	<0.001
**I-BET-762**	0.005	**Umbralisib (TGR-1202**)	0.005	1400W 2HCl	0.009
Tucidinostat (Chidamide)	0.002	AZD1480	0.004	Fenretinide	0.025
Dapivirine (TMC120)	0.021	Enoxacin Sesquihydrate	0.001	Clodronate Disodium	0.066
AT101	0.009	Resiquimod	0.003	IOWH032	0.010
Betaine	0.031	LXR-623 (WAY-252623)	0.001	PX-478 2HCl	0.016
AT9283	0.046	Ivosidenib (AG-120)	0.001	AZD6738	0.064
PND-1186 (VS-4718)	0.043	AMG319	0.004	PRI-724	0.068
VGX-1027	0.005	Encorafenib (LGX818)	0.006	Sodium dichloroacetate (DCA)	0.037
PF-00562271	0.011	Propofol	0.001	Pterostilbene	0.063

Δ (subtype vs rest)

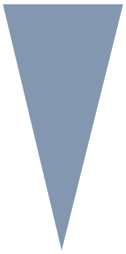

Figure S5. Ranking of the top ten most subtype specific compounds based on differential cell viability assessed by the focused in-house HCS. Δ = mean percentage of viable cells of ‚rest‘ – mean viable cells of subtype ‚x‘. Hits used for further validation are marked in bold.

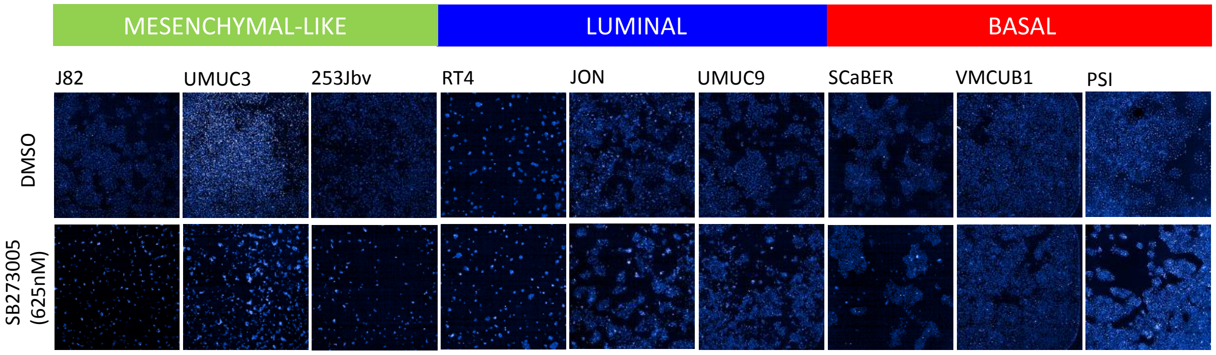

Figure S6. Representative images of 9 cell lines after 72h incubation with 625nM of the integrin inhibitor SB273005. Blue = cell nuclei stained with Hoechst 33342.

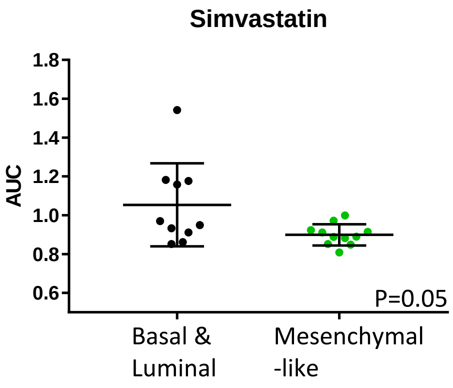

Figure S7. Differential response of CCLE cell lines to Simvastatin assessed by the unpaired two-tailed Student's t-test with Welch correction. (p=0.05)

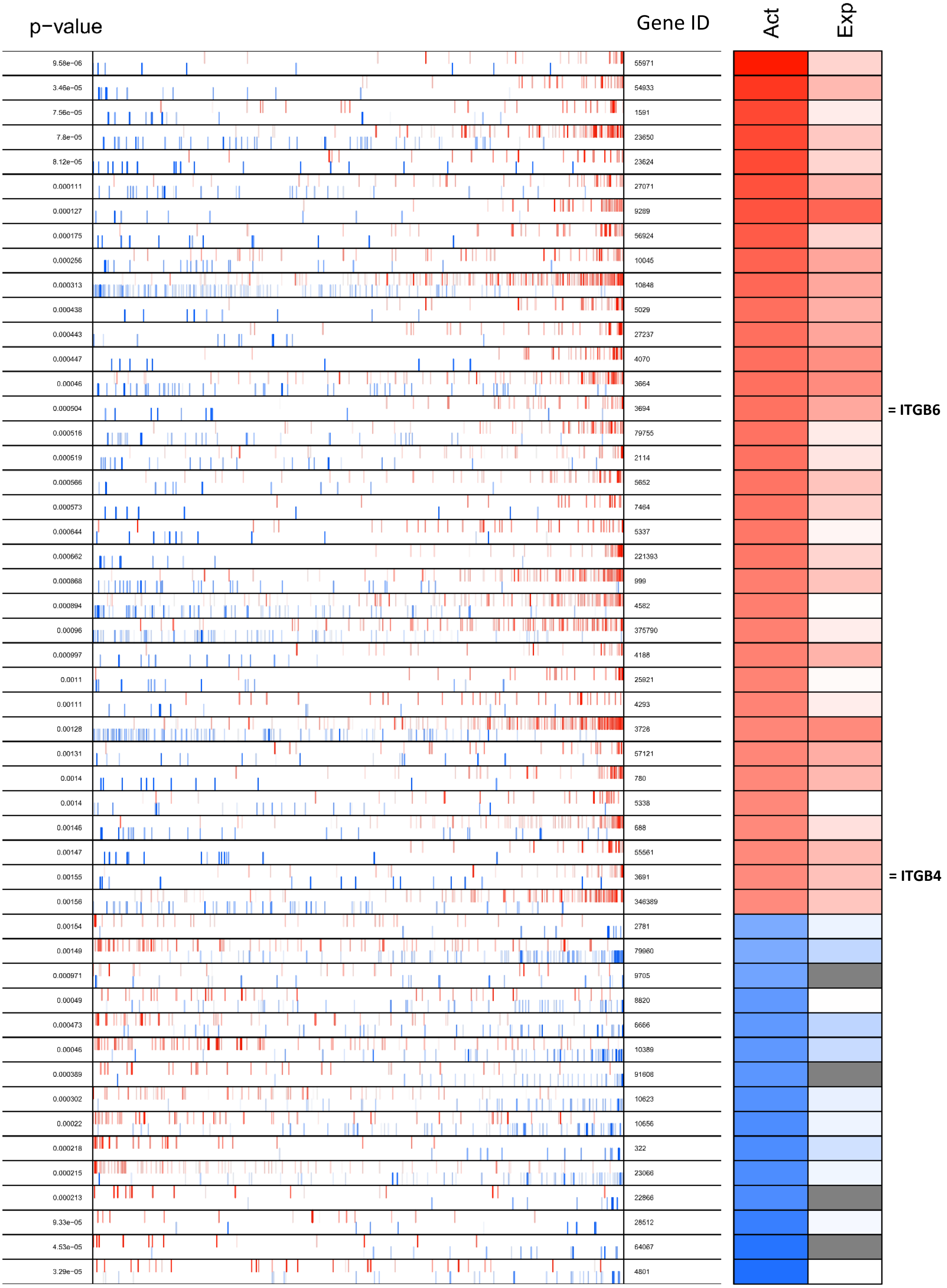

Figure S8. Virtual Inference of Protein-activity by Enriched Regulon analysis (VIPER) displaying differential regulon activity (first column. red = activated. blue = inactivated) and regulon expression (second column. red = high expression. blue = low expression. gray = not applicable) of the mesenchymal-like CCLE cell lines. Representation of the top 50 most significantly activated or inactivated regulons.

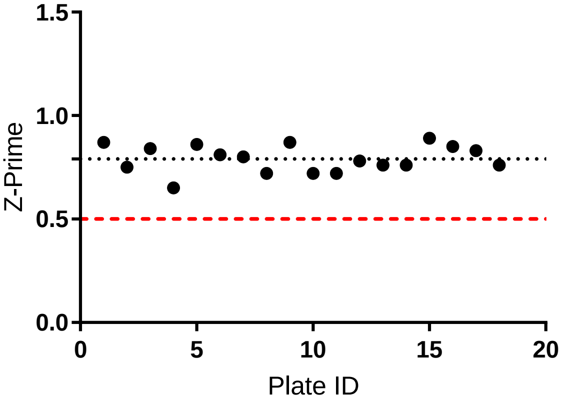

Figure S9. Z-prime scores of the focused HCS plates, screening 9 preselected BLCA cell lines incubated with 616 clinical and preclinical drugs.
